# Genomic, Microbial and Immunological Microenvironment of Colorectal Polyps

**DOI:** 10.3390/cancers13143382

**Published:** 2021-07-06

**Authors:** Benita C. Y. Tse, Zoe Welham, Alexander F. Engel, Mark P. Molloy

**Affiliations:** 1 Bowel Cancer and Biomarker Laboratory, Faculty of Medicine and Health, School of Medical Sciences, The University of Sydney, Sydney 2006, Australia; benita.tse@sydney.edu.au (B.C.Y.T.); zwel5671@uni.sydney.edu.au (Z.W.); 2 Department of Colorectal Surgery, Royal North Shore Hospital, Sydney 2065, Australia; alexander.engel@sydney.edu.au; 3 Faculty of Medicine and Health, Northern Clinical School, The University of Sydney, Sydney 2006, Australia

**Keywords:** colorectal polyps, adenoma, colorectal carcinoma, microbiome, immunogenic, mutational burden, colonoscopy

## Abstract

**Simple Summary:**

Colorectal cancers (CRC) initiate from small cell clusters known as polyps. Colonoscopic surveillance and removal of polyps is an important strategy to prevent CRC progression. Recent advances in sequencing technologies have highlighted genetic mutations in polyps that potentially contribute to CRC development. However, CRC might be considered more than a genetic disease, as emerging evidence describes early changes to immune surveillance and gut microbiota in people with polyps. Here, we review the molecular landscape of colorectal polyps, considering their genomic, microbial and immunological features, and discuss the potential clinical utility of these data.

**Abstract:**

Colorectal cancer (CRC) develops from pre-cancerous cellular lesions in the gut epithelium, known as polyps. Polyps themselves arise through the accumulation of mutations that disrupt the function of key tumour suppressor genes, activate proto-oncogenes and allow proliferation in an environment where immune control has been compromised. Consequently, colonoscopic surveillance and polypectomy are central pillars of cancer control strategies. Recent advances in genomic sequencing technologies have enhanced our knowledge of key driver mutations in polyp lesions that likely contribute to CRC. In accordance with the prognostic significance of Immunoscores for CRC survival, there is also a likely role for early immunological changes in polyps, including an increase in regulatory T cells and a decrease in mature dendritic cell numbers. Gut microbiotas are under increasing research interest for their potential contribution to CRC evolution, and changes in the gut microbiome have been reported from analyses of adenomas. Given that early changes to molecular components of bowel polyps may have a direct impact on cancer development and/or act as indicators of early disease, we review the molecular landscape of colorectal polyps, with an emphasis on immunological and microbial alterations occurring in the gut and propose the potential clinical utility of these data.

## 1. Introduction

Colorectal cancer (CRC) is the second most common cancer in females and third most common in males. Currently accounting for 10% of all new cancer diagnoses a year and 700,000 deaths worldwide, CRC incidence is expected to continue to increase [1]. It has been well established that the development of sporadic CRC begins with precursor growths in the lining of the bowel epithelium, known as polyps. Neoplastic polyps harbor malignant potential due to loss of function of tumour suppressor genes and/or activation of driver proto-oncogenes [2].

In sporadic CRC, two major genetic pathways underlying CRC progression from polyps have been described: (i) the conventional adenoma–carcinoma pathway, and (ii) the serrated neoplasia pathway. The conventional adenoma pathway accounts for approximately 70% of CRCs and is defined by loss of function mutation in the Wnt/beta catenin pathway regulator gene *APC*, typically followed by activating mutations of the *KRAS* oncogene, leading to a chromosomal instability (CIN) genotype of microsatellite stable CRC [1]. Histologically, tubular adenoma, tubulovillous adenoma and villous adenoma are subtypes genetically driven through the conventional CIN pathway, they and represent the most frequent polyps found in the colorectum. In comparison, the alternative genetic route is known as the serrated neoplasia pathway that accounts for the remaining ~30% of CRC. Within this pathway, sessile serrated adenoma/polyps (SSA/P) are characterised by *BRAF* mutations and high levels of CpG island methylator phenotype (CIMP) [3,4]. Epigenetic silencing of *MLH1* in some SSA/P results in deficient mismatch repair and high microsatellite instability (MSI-H), allowing for the accumulation of numerous advantageous mutations that facilitate dysplasia and carcinogenesis. Notably, epigenetic silencing of tumour suppressor genes other than *MLH1* can occur in SSA/P, resulting in CIMP-high, microsatellite stable CRCs. SSA/P are observed with greater frequency in the proximal colon. Traditional serrated adenomas (TSA) are lowly prevalent, genetically heterogeneous lesions typically observed in the distal colon and rectum. TSAs can harbor *KRAS* or *BRAF* mutations, or neither, and can be CIMP-low or high, and are microsatellite stable. *MLH1* is rarely under epigenetic silencing in TSA, although *MGMT* may be hypermethylated, eliminating the repair of adducted DNA. [3,5,6].

MicroRNA (miRNA) are small, single-stranded non-coding RNAs that contribute to health and disease through post-transcriptional gene regulation. miRNAs have been identified to play key roles in CRC carcinogenesis through regulating a plethora of genes involving oncogenic signaling pathways, including Wnt, Ras, transforming growth factor beta (TGF-β) and NF-κB/AKT/STAT3, as well as other regulatory pathways such as stemness, epithelial–mesenchymal transition and metastasis [7,8,9,10,11,12,13,14,15,16,17]. Readers are referred to reviews describing how miRNA contributes to CRC carcinogenesis [18,19,20].

**Figure 1:**
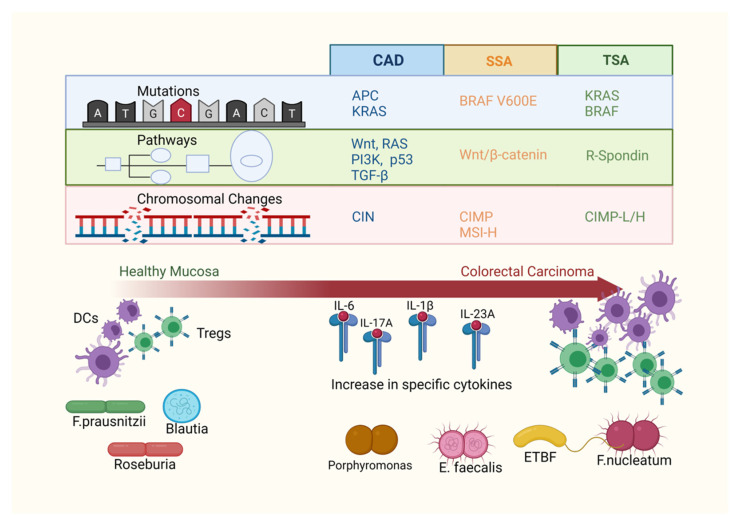
The progression of colorectal polyps to adenocarcinoma is multi-factorial, with different polyp subtypes showing patterns in their genetic, immune and microbiome features. Genetic environment: conventional adenomas (CAD) are characterised by chromosomal instability (CIN) and show early mutations in the adenomatous polyposis coli (*APC*) and *KRAS* genes, with dysregulated Wnt, RAS, PI3K, p53 and TGF-β intracellular pathways. Commonly driven by *BRAF V600E* mutation and with disruptions in Wnt/β-catenin signalling pathways, serrated sessile adenomas (SSAs) show aberrant methylation, (CIMP+) and can be MSI-high or MSS. Traditional serrated adenomas (TSAs) can have either *BRAF* or *KRAS* mutations, *RNF43* inactivation and often show *R-spondin* gene fusions. They may be either CIMP-low or CIMP-high. Immune environment: progression from polyps to carcinoma is correlated with a decrease in the ratio of mature dendritic cells to immature dendritic cells (DCs), as well as an increase in the relative number of regulatory T cells (Tregs). Increases in pro-inflammatory cytokines such as IL-6, IL-17A, IL-1β and IL-23A are reported. Microbiome environment: relative to healthy tissue, mucosal polyps and carcinoma have altered microbial communities such as decreases in *F. prausnitzii, Roseburia* and *Blautia*, and an increased relative abundance of bacteria including enterotoxic *Bacteroides fragilis* (ETBF), *E. faecalis* and *F. nucleatum*.

The major genetic alterations underlying conventional adenomas and serrated lesions are now well-established. Next generation sequencing (NGS) is enabling even deeper discovery of the mutational profiles within polyp subtypes. However, CRC is more than a genetic disease, and understanding how microbial and immune activity contributes to CRC’s progression at the pre-malignant polyp stage is important to gain a more holistic understanding and identify new prevention and treatment strategies. The purpose of this review is to summarise our understanding of the genomic burdens, immune activity and microbiomes associated with sporadic colorectal polyps (Figure 1).

## 2. Gene Mutations in Colorectal Polyps

Advancements in NGS have revealed detailed new molecular data, cataloguing the key driver and passenger mutations associated with colorectal adenocarcinoma and their precursor adenoma and SSA/P lesions. Nikolaev and colleagues examined early CRC carcinogenesis, and their seminal work using whole-exome sequencing of 24 colorectal adenomas demonstrated that these pre-cancers exhibited a ‘mutator phenotype’ of high, single nucleotide substitutions compared to non-transformed cells, which was enabled through inactivation of a gene responsible for genomic stability, proposed to be *APC* [21]. Consistent with the Vogelstein model of stepwise accumulation of driver mutations in the transition of conventional adenoma to adenocarcinoma, Zhou and colleagues applied exome capture sequencing of 73 pairs of matched adenoma and adenocarcinoma samples, showing higher numbers of nonsynonymous somatic single nucleotide variations in adenocarcinomas compared to adenomas [22]. Whole-exome sequencing and targeted sequencing of 149 polyp samples showed that serrated adenomas and conventional adenomas had similar frequencies of somatic mutations, but with distinctive driver mutations consistent with their differing genetic origins [23]. Combined, exome-wide studies of conventional adenomas reveal that *APC, KRAS, PIK3CA, TP53* and *SMAD4* genes affecting Wnt, RAS, PI3K, p53 and TGF-beta signaling pathways are amongst those most commonly altered, often with significantly increasing mutation prevalence in development from non-advanced conventional adenomas to advanced conventional adenomas to CRC [23,24,25,26,27,28].

In contrast to conventional adenomas, *BRAF* V600E is the most highly prevalent driver for SSA/P neoplasia. In a study of 234 polyps, *BRAF* mutations were detected in 81–92% of SSA/P subtypes and only 0–9% of these lesions had *KRAS* mutations. *KRAS* and *BRAF* mutations were always mutually exclusive [29]. Mutational inactivation of the E3 ubiquitin-protein ligase *RNF43* is common in SSA/P, and often co-occurs with *BRAF* and is absent in tubulovillous/villous adenoma [30,31]. However, the mutational frequency of *RNF43* inactivation in sporadic disease varies across studies, serrated lesion subtype and degree of dysplasia [32]. Similar variation has been reported for *APC* in SSA/Ps (low in SSA/P without dysplasia > dysplastic SSA/P > TSAs) [33,34]. TSA are the least common type of colorectal serrated polyp and show considerable molecular diversity. Sequencing of 128 TSAs showed 97% harboured mutations in MAPK pathway genes (predominantly *BRAF* V600E or *KRAS*) and 84% had mutations in WNT pathway genes [35]. Among WNT pathway genes, R-Spondin (*RSPO*) gene fusions and overexpression is frequently observed [36].

## 3. Tumour Mutational Burden

The growing ease and rapidity of NGS molecular profiling has grown the idea of tumour mutational burden (TMB) as a facile clinical biomarker, especially for predicting response to immunotherapies [37]. The field is yet to settle on a standard for measuring TMB, with growing interests evaluating use of more cost-effective panel sequencing in place of whole exome sequencing (WES) [38]. Additionally, approaches to mutation calling and TMB quantification remain challenging in samples with low tumor content, while defining cut-points for discriminating high versus low TMB appears to be study-specific. [39]. Nonetheless, in advanced melanoma, non-small cell lung carcinoma, urothelial cancer, renal cancer and others, high TMB is commonly reported to be predictive for response to immune checkpoint immunotherapy and improved overall survival (OS) [40,41,42,43,44,45,46,47]. In CRC, patients with deficient mismatch repair, MSI-H tumours showing high TMB and treated with the programmed cell death protein 1 (PD-1) inhibitor pembrolizumab as first-line monotherapy showed significantly improved progression-free survival compared to standard of care chemotherapy with or without bevacizumab or cetuximab [48]. In a trial evaluating the benefit of adding bevacizumab or cetuximab to oxaliplatin- or irinotecan- based chemotherapy, microsatellite stable (MSS) colorectal tumours classified as high TMB (>8/Mb) compared to low TMB achieved limited, but longer median overall survival (33.8 months versus 28.1 months), while no differences were observed between treatment arms. It was hypothesised that higher TMB tumours would possess increased lymphocyte infiltration, and this may be a factor in the survival outcomes [49]. As a prognostic factor, the clinical utility of TMB in different cancer types is still to be resolved. Nonetheless, several studies using different methodologies for determining TMB and high/low cut-points have reported that higher TMB is associated with worse survival for colorectal cancer patients who do not receive immune checkpoint inhibitors [50,51,52].

The clinical utility of measuring mutational burdens in the pre-cancer setting of colorectal polyps is yet to be established. Nonetheless, several emerging studies mentioned below provide data to investigate this prospect. WES of 25 colorectal adenomas and 10 matched adjacent mucosas from familial adenomatous polyposis patients reported a mutation frequency of 1.75 mutations per Mb of exome sequence in polyps, while non-hypermutated adenocarcinomas had a mean of 4.26 mutations/Mb [53]. Lin and colleagues used WES to establish an average non-silent somatic mutation rate of 1.6/Mb for sporadic adenomas compared to 4.6/Mb for non-hypermutated CRC samples in The Cancer Genome Atlas (TCGA) database [23]. Non-significant differences in mutational burden were reported when comparing conventional adenomas and SSA/Ps (1.5 and 1.7/Mb, respectively). In this cohort, small differences were reported between non-advanced and advanced adenomas (1.6 and 2.0/Mb, respectively). More recently, an examination of 58 large conventional adenomas with low and high grade dysplasia, using an alternative approach of panel sequencing of 409 cancer genes, reported a mean nonsynonymous variant rate of 8.0/Mb compared to 8.8/Mb for these genes in adenocarcinomas from the TCGA CRC dataset [54]. These studies demonstrate that TMB differences between polyps and adenocarcinomas are observed using either WES or gene panel sequencing. What is currently lacking is an assessment of whether the variations in mutational burden and mutation patterns seen within adenomas reflect functional differences, such as malignancy risk or metastatic potential, either of which could have important clinical value. It is likely that polyps of clinical concern would be the TMB outlier cases (i.e., adenomas with TMB similar to those measured for CRCs). Perhaps the combination of histological findings and polyp mutational burdens would contribute to a more informative prognostic risk score by taking into consideration mutations in oncogenic drivers. This score is missing in current clinical practice risk assessments and might be informative for the timing of surveillance colonoscopies.

## 4. Immunological Environment within Polyps

Cancer development involves continuous cross-talk between malignant cells and the immune system in a process known as cancer immunoediting. The adaptive immune system eliminates highly immunogenic tumours [55,56] and controls tumour cells with lower immunogenicity until spontaneously advantageous mutations allow for escape from immune control [57]. Thus, the type, function, density and location of immune cells within tumour regions have been extensively studied. In CRC, this concept enabled the establishment of the ‘Immunoscore’, whereby cancers are graded based on the densities of pan-T cells and cytotoxic T cells [58,59]. CRC patients with high densities of lymphocyte infiltrate have increased overall survival and reduced tumour recurrence [60,61]. Despite the Immunoscore being a better predictor of CRC recurrence and survival than histopathological features [60], relatively little is known about the immune contexture of pre-cancerous colorectal polyps.

The immunological environment within colorectal polyps is largely driven by the developmental pathways. SSA/Ps with microsatellite instability (MSI) have high accumulated mutational burden and appear to be highly immunogenic. Acosta-Gonzalez and colleagues showed that the sequential progression of SSA/Ps through low-grade to high-grade dysplasia was paired with increasing density of intraepithelial lymphocytes and increasing expression of checkpoint inhibitors PD-1 and its ligand PD-L1 [62]. In comparison to cancers arising from conventional adenomas, cancers arising from SSA/Ps have greater numbers of tumour-infiltrating lymphocytes (TILs), at least in part supported by a high number of Crohn’s-like reactions. These TILs act as local tertiary lymphoid structures that facilitate lymphocyte recruitment and expansion [63]. Elevated TILs and Crohn’s-like reactions are associated with MSI. Murakami et al. note their observations are consistent with a tumour-related immune response in SSA/Ps, where malignancy develops in the presence of amplified TILs and Crohn’s-like reactions.

Immunohistochemical analyses reveal that conventional adenomas have decreased numbers of mature dendritic cells whilst showing increasing numbers of immature dendritic cells compared to mucosa of healthy controls [64]. The reduction in number of mature dendritic cells continues as conventional adenomas progress to cancer [64]. As mature dendritic cells are responsible for presenting antigens, as well as activating adaptive immune cells, the lack of mature dendritic cells suggests an inability to generate tumour antigen-specific immune responses. This observation is consistent with a reduction in the expression of antigen presentation molecule, human leukocyte antigen-DR (HLA-DR), in both the epithelium and stroma of colorectal carcinomas compared to matched adenomas [65]. A decrease in HLA-DR expression in the stroma was also observed in the progression from early stage to late-stage carcinomas, which may have wider implications for immunotherapeutic development.

Similarly, other signs of a dampened immune response can be observed in conventional adenomas. An increase in mucosal cyclooxygenase-2 (COX-2) mRNA has been reported in the progression of colorectal adenomas to cancers [64]. Activation of COX-2 leads to the production of immune suppressive molecules such as prostaglandin E2. COX-2 expression and HER-2 have previously been described as important regulators of CRC invasion and metastasis [66]. In addition to COX-2, the mRNA expression of costimulatory molecules CD80 and CD86 was altered in polyp patients compared to control patients. Required for effective activation of T cell receptors and subsequent cell-mediated immunity, the expression of CD86 was higher within polyp tissue whilst CD80 expression was reduced [67].

Regulatory T cells accumulate within colorectal tumours and are immune suppressive by inhibiting the transepithelial migration [68,69], activation [70] and proliferation of effector T cells [71]. Together, they suppress the anti-colorectal tumour immune response in a COX-2-dependent manner [72]. The number of intraepithelial regulatory T cells was observed to increase when adenomatous polyps with low-grade dysplasia were compared to advanced adenomas and CRCs [73,74]. Interestingly, this accumulation appears specific to sporadic adenomas and has not been reported in familial adenomatous polyposis [75]. The accumulation of regulatory T cells in colorectal adenomas was equivalent to CRCs and associated with an increase in expression of FoxP3 and IL-10 [76]. Furthermore, the increase in regulatory T cells could be observed in peripheral blood of patients with colorectal polyps (either serrated adenomas or conventional adenoma polyps), whereby regulatory T cells consisted of 2.9% of all circulating lymphocytes [77]. This is higher than patients without polyps (at 1.8%) but lower than patients with CRC (at 12.6%). However, this is at least in part due to an overall increase in peripheral helper T cell frequency during CRC progression [77]. In addition to changes in peripheral frequencies, it should be noted that regulatory T cells from polyp patients and CRC patients showed varying expressions of some genes. In particular, only CRC peripheral regulatory T cells expressed the immune suppressive cytokine IL-10, whilst only polyp regulatory T cells had significantly lower expression of the IL-10 receptor α [77]. This suggests that regulatory T cell function changes throughout CRC progression.

There remain many immune cell subsets whose presence in, and/or influence on, colorectal polyps is not well defined (Table 1). For example, whilst regulatory T cells have been explored, less is known about other subsets of T cells. However, studies investigating cytokine expression hint at the associated phenotype changes. IL-17A is a cytokine typically produced by Th17 cells, and an increase in the expression of IL-17A can be seen in the adenoma–carcinoma sequence [78]. In particular, IL-17A mRNA levels in colorectal adenomas increase with the severity of dysplasia and can be detected in adjacent tissues [78,79]. In combination with an associated increase in its receptor, IL-17 receptor A [80], and other Th17-inducing cytokines such as IL-23A, IL-1β and IL-6 in colorectal adenomas [79], Th17 cells may have an important role in defining the immune microenvironment in early CRC development. Colorectal adenomas also show increased expression of Th1-associated cytokines (IFN-γ, TNF-α, IL-12A, IL-18) compared to normal mucosa and CRC [81]. More in-depth analyses of T cell subsets within colorectal adenomas are warranted in the future, especially given that CRC patients with higher expression of Th1-related genes show prolonged disease-free survival, whilst patients with high expression of Th17-associated genes have poorer prognoses [82]. Hence, the Th1/Th17 ratio within colorectal adenomas may be an important early prognostic indicator. Indeed, these cytokine changes are not only local but can be seen in the serum, where systemic increases in IFN-γ, IL-4, IL-5 and IL-12 have been reported in colorectal adenoma patients compared to controls [83]. In particular, the increases in serum IL-4 are consistent with CRC patients, whereby increases are associated with higher CRC staging [84]. This suggests that some of these T cell changes are systemic immune responses to colorectal adenomas, but this requires further investigation.

Similarly, many innate immune cells are known to be altered in CRC and/or observed in mouse models (e.g., APC^min/+^), but are poorly explored in human polyps. Investigations into macrophages within colorectal polyps are warranted given that high macrophage infiltration is associated with improved survival [85] and reduced liver metastasis in CRC [86]. Neutrophil infiltration on the tumour front also indicates better patient prognosis in Stage I-II CRC [87]. Conversely, CRCs have reduced numbers of natural killer cells in comparison to normal mucosa, and those patients with high NK tumour infiltration achieve better overall survival [88,89]. Mouse models of colorectal adenoma and carcinoma offer insight into the importance and influence of myeloid cells in polyp development. In the APC^min/+^ model of FAP, Stat6 was found to be a mediator of polyp development, at least in part by promoting the expansion of myeloid-derived suppressor cells (MDSCs) in the lamina propria [90]. In the same model, the chemokine CCL2 was found to promote the accumulation and immune suppressive capabilities of MDSCs in polyps [91]. The authors also noted increases in CCL2 levels within the colitis-associated CRC and adenomas of patients, suggesting that CCL2 may play a role in human CRC development. Similarly, invariant natural killer T (iNKT) cells are reduced in number within APC^min/+^ mouse polyps [92]. Deletion of iNKT in these mice or reducing iNKT cell frequency by treatment of cell ligands led to a reduction in polyp burden [92,93], again suggesting a role of iNKTs in polyp development. It is important to note, however, that sporadic colorectal adenomas in humans are genetically complex and heterogeneous (as mentioned above). Hence, the APC^min/+^ model offers insight, but not necessarily evidence, for the role of these myeloid cells in human colorectal adenoma.

Given that the location of immune cells within the cancer environment and their interaction with other immune cells dictate the immune response and the overall survival of patients [94], more in-depth analyses of immune cell subsets in the early stages of CRC development may have important prognostic value. A major limitation of previous investigations has been the one to two marker immunohistochemical stains for immunophenotyping; this limitation restricts the ability to accurately define subsets and immune cell interactions. Therefore, use of newer multiplexed capabilities will allow more in-depth phenotyping of immune cells, which is required to gain a deeper understanding of their frequency and role in the progression of CRC.

**Table 1:** Comparison of known immune cell associations with human polyps and CRC.

Immune Cell Subset	Associations in Polyps	Associations in CRC
T cells		T cell numbers in the tumour centre are associated with improved disease-free survival [95].
Cytotoxic T cells		Cytotoxic T cells in the tumour centre are associated with improved disease-free survival and overall survival [95,96].
Helper T cells		High expression of Th1-related genes is associated with increased disease-free survival, whilst a high expression of Th17-related genes is associated with poor prognosis [82].
Regulatory T cells	Increases in number with malignant transformation [73,74].	Increase in number in tumour compared to normal mucosa [97]. High infiltrate within the tumour stroma and centre is associated with improved overall survival [95,96].
Mucosa-associated invariant T cells		Accumulate in tumour tissue irrespective of stage [98].
Natural killer T cells		Increase in number in CRC. Patients with high natural killer T cell infiltration associated with better overall survival [99].
B cells		Higher numbers of infiltrating B cells seen in tumours of the right colon and associated with better disease-free survival [100].
Dendritic cells	Reduced numbers of mature dendritic cells and increased numbers of immature dendritic cells seen in conventional adenomas [64].	Infiltration of dendritic cells is higher in MSI-H lesions [101]. Improved survival of patients with high infiltration of dendritic cells [102].
Macrophages		High macrophage infiltration is associated with improved survival in colon cancer and reduced liver metastasis [85,86].
Neutrophils		High neutrophil infiltration in the tumour front is associated with better prognosis in patients with stage I-II CRC [87].
Natural killer cells		CRCs contain fewer natural killer cells than adjacent normal mucosa [88]. Patients with extensive natural killer cell infiltration have higher rates of overall survival [89].

## 5. Microbiota and Colorectal Polyps

Gut microbiota represent a diverse ecosystem consisting of several trillion bacteria that perform important health functions for the host. Species composition between individuals is varied [103], which may be due to host genetics, diet, exercise, alcohol and smoking habits, as well as early life microbial exposure [104,105,106]. Microbiota in the human gut are predominantly anaerobes, with the two phyla Firmicutes and Bacteroidetes comprising 90% of the community, and the phyla Actinobacteria, Proteobacteria, Fusobacteria and Verrucomicrobia comprising the majority of the remainder [107]. Through improvements to analytical technologies, it has become evident that changes to the gut microbiome are observed along the axis of CRC carcinogenesis: healthy-neoplastic-malignant mucosa [108]. Most studies investigating the gut microbiome use faecal analysis as a convenient proxy for sampling the bowel, while fewer studies directly measure colonic mucosal microbiota. This is an important distinction, as mucosal microbiota directly interacts with mucins and receptors on the cell surface at specific gut locations, while microbiota in stools represent those transported in the intestinal lumen [109,110]. Given that some bacterial pathogens have toxins that can cause oncogenic transformation (e.g., pks^+^ *E. coli*) [111], and others have been commonly localised with colorectal tumours (i.e., *Fusobacterium* spp.), it is of interest to have wider knowledge of the microbes associated with neoplastic colorectal lesions.

One of the first studies to examine bowel–polyp-associated microbiota [112] used terminal restriction fragment length polymorphism (T-RFLP) to compare normal rectal mucosal biopsies from patients with and without adenomas. Moreover, 16S rRNA sequencing of four adenoma and four controls (non-polyp patients) showed enrichment of Proteobacteria and depletion of Bacteroidetes in adenomas compared to controls, while the phylum Firmicutes was similar in both groups. However, amongst the Firmicutes, adenoma cases showed a relative increase in *Faecalibacterium* spp. and *Dorea* spp., and a relative decrease in *Bacteroides* spp. and *Coprococcus* spp.

Magnifesta and colleagues [113] directly compared polyp mucosa with healthy marginal tissue from 12 polyp cases consisting of proximal tubular adenomas and rectal hyperplastic polyps. They found no overall changes at the phylum level, but they did observe enrichment of specific bacteria such as *Lactobacillus*, *Helicobacter* and *Klebsiella*, and a relative depletion of bacteria, including *Bifidobacterium*, *Faecalibacterium*, *Eschericia-Shigella*, *Bacteroides*, *Blautia* and *Lachnoclostridium*, in polyp mucosa compared to adjacent healthy tissue. Similar results were reported by Sanarpeddy and colleagues [114], with relative increases in *Lactobacillus* and *Helicobacter*, in addition to other genera, in 33 adenoma cases compared to controls. Recently, Wang et al. [115] compared biopsy samples from 49 advanced adenomas (AA) (mean size > = 10 mm) with adjacent normal mucosa, and 36 healthy control samples. AA mucosa showed enrichment of Proteobacteria phyla and depletion of Firmicutes and Bacteroidetes phyla compared to healthy controls. More specifically, bacteria from the *Oceanospirillales* family and *Shewanella* algae showed enrichment, while *Blautia*, *Coprococcus*, *Bacteroides* and *Faecalibacterium prausnitzii* were depleted. Contrary to other reports, *Fusobacterium* were depleted in AA cases compared to healthy controls.

Dadkhah and colleagues [116] used a machine learning approach to compare 138 mucosa, 183 stool and 231 rectal swab samples of adenoma cases (including non-neoplastic polyps, benign (50%), advanced (2%) and high-risk adenomas (21%)) with healthy controls. Overall, they reported a lower abundance of Proteobacteria in polyp cases compared to controls. In addition, the genera *Bifidobacterium*, *Faecalibacterium* and *Blautia* were depleted in adenoma mucosa samples compared to control mucosa but, at the species level, there was no enrichment of the pathogens *Fusobacterium nucleatum* and *B. fragilis*.

A study comparing the microbiome and metabolome reported significant enrichment of *Bifidobacterium*, as well as a trend towards enrichment for *Escherichia coli*, *Clostridium* and *Bacteroides*, in 15 adenoma cases compared to 15 healthy controls [117]. Moreover, 23 metabolites were associated with differences between adenoma and control cases. In particular, there was an increase in inflammatory metabolite prostaglandin E2 and a decrease in antioxidant metabolites 5-oxoproline and diketogulonic acid in adenoma cases compared to controls. These metabolites were associated with inflammatory responses, carbohydrate metabolism and GI disease pathways.

*Fusobacterium nucleatum*, a notable oral microbe, has previously been indicated in colorectal pathogenesis [118]. McCoy and colleagues [119] used qPCR to determine that *F. nucleatum* was significantly enriched in the rectal mucosa of 48 adenoma patients compared to 67 healthy controls. This agrees with Kostic and colleagues [120], who reported enrichment of *Fusobacterium* spp. in adenomas relative to adjacent tissues, and in stool samples from colorectal adenoma and carcinoma patients compared to healthy subjects. Ito and colleagues [121] also used qPCR to compare *Fusobacterium* in formalin-fixed paraffin-embedded (FFPE) tissues of 343 sessile serrated adenomas, 122 conventional adenomas and 511 CRC cases. *F. nucleatum* was commonly detected but could not sufficiently differentiate between these pathologies: 24% of HPs, 35% of SSAs, 30% of TSAs and 33% of non-serrated adenoma showed high *F. nucleatum*. In particular, *F. nucleatum* was more frequent in CIMP-high lesions and was positively correlated with grade. The inability of *F. nucleatum* to parse differences between SSA and conventional adenomas was also reported by Yoon [122]; however, with only six samples per group, these findings need clarification with sufficient statistical power.

In summary, the above studies demonstrate that, like CRC, colorectal adenomas commonly harbor an altered microbiome compared to healthy mucosa. Whether this is cause or effect remains a matter of much debate [123,124]. Key findings from analyses of bowel polyp mucosa (Table 2) suggest a relative enrichment of bacteria from the phylum Proteobacteria, particularly the orders Pseudomonodales (e.g., genus *Pseudomonas*), Enterobacterales (e.g., genera *Escherichia-Shigella*, *Klebsellia*) and Campylobacterales (e.g., genus *Helicobacter*) in adenoma cases compared to controls. There is also relative enrichment from the phylum Firmicutes, predominantly class Bacilli (e.g., *Lactobacillus* and *Lactococcus*), in adenoma cases compared to controls. Consistent with the previous literature, *Fusobacterium* species were often found to be enriched in adenoma cases compared to controls. In contrast, some Firmicutes bacteria tend to be depleted in adenoma cases compared to controls, particularly from class Clostridia (e.g., *Blautia*, *Faecalibacterium* and *Bacilli*). This is also the case with genera from phyla Actinobacteria (e.g., *Bifidobacterium*) and Bacteroidetes (e.g., *Bacteroides*). However, it must be noted that reports were not always consistent. For example, some studies showed a trend towards depletion in bacteria in adenoma cases from Fusobacteria taxa, as well as from phylum Proteobacteria (e.g., *Escherichia coli*, *Escherichia-Shigella*) and Firmicutes (e.g., *Lactobacillus*) that were commonly reported to be enriched in other studies.

Although these studies highlight some recurrent and differentially abundant species, they also report many bacteria that are specific for each study. This may be influenced by large inter-individual microbiome composition differences at the genera and species levels [125], or may be an artefact of the different bioinformatic analysis pipelines employed. Either way, the choice of 16S rRNA primer selection as a limitation to reliably discriminate bacteria at the species level [126] could contribute to differences in bacterial taxonomy assignment. However, this variability in identified species also highlights the importance of understanding their functional role, as the interplay between bacteria and metabolite production is complex [127]. Both human and mouse models suggest that the adenoma microbiome is characterised by a decrease in butyrate-producing bacteria (e.g., *Faecalibacterium prausnitzii*), which show a protective effect on gut permeability and inflammation and an increase in bacteria that increase epithelial permeability and inflammation, produce reactive oxygen species and damage DNA [128]. It is not well understood which bacteria induce these early processes. A more comprehensive approach, currently missing from studies in bowel polyps, would be to utilise whole genome shotgun metagenomic sequencing, which can incorporate functional analysis with species-level identification. Together with larger sample sizes for increased statistical power, this approach may clarify which bacteria are involved in these processes.

## 6. Interaction of Microbiota and the Immune System in CRC Tumorigenesis

Microbiota have a role in the development and regulation of the host immune system, including regulating inflammation, which is associated with increased risk of CRC [131,132,133]. The intersection of microbes and immune-regulated mucosal inflammation is linked to bowel tumorigenesis. For example, mouse studies have shown the importance of CD4+ helper T cells and Foxp3+ Tregs secretion of IL-10 to control intra-polyp inflammation and growth in a polyposis-prone APC disruption model [134]. Depleting IL-10 resulted in a fivefold increase in colonic polyps and alterations to epithelial barrier integrity that enabled the expansion of pathogenic bacteria Bacteroides and Porphyromonas within colonic polyps compared to healthy mucosa. Broad spectrum antibiotics attenuated inflammation and polyposis, establishing a clear link between microbes, inflammation and tumouriogenesis. Using germ-free APC^min/+^; IL10^−/−^, Tobin and colleagues found alternative microbes in stool that positively correlated with increased tumour numbers in mice exposed to specific pathogen-free bacteria, namely Akkermansia, Blautia, Dorea, Enterococcus and Escherichia/Shigella; however, altered levels of Bacteroides and Porphyromonas were not found [135]. Interestingly, there was no correlation with bacteria and tumourigenesis in APC^min/+^ mice that retained IL-10 secretion, underscoring the importance of immune–microbiota interactions. *Enterococcus faecalis*, in addition to promoting superoxide and hydrogen peroxide-induced DNA damage [136], is associated with increased NF-κB-mediated pro-inflammatory cytokine (IL-6 and IP-10) gene expression in IL-10-deficient mice [137]. Enterotoxic *Bacteroides fragilis* (ETBF), in addition to inducing bowel mucosa permeability [138], can induce carcinogenesis in APC^min/+^ mice through the activation of a Stat3-driven TH17 response, with the blockade of IL-17 resulting in the inhibition of ETBF-induced tumours [139]. ETBF also enhanced Treg-initiated IL-17-mediated carcinogenesis in C57BL/6 FOXP3^DTR^ mice [140]. In addition to its activity as an oncomir [141], and utilising its virulence factor FadA to infiltrate epithelial cells and activate proliferation pathways [142], *F. nucleatum* can recruit tumour-infiltrating immune cells in APC^min/+^ mice, resulting in upregulated pro-inflammatory genes [120]. *F. nucleatum* can also inhibit T cell activation and NK-mediated killing of tumour cells [143]. Other bacteria indicated in inflammation-mediated colon carcinogenesis include *Streptococcus gallolyticus* [144] and pks+-harbouring *Escherichia coli*, which are indicated in ROS production and induce macrophage COX-2 and prostaglandin E2 pro-carcinoma activities [145].

**Figure 2:**
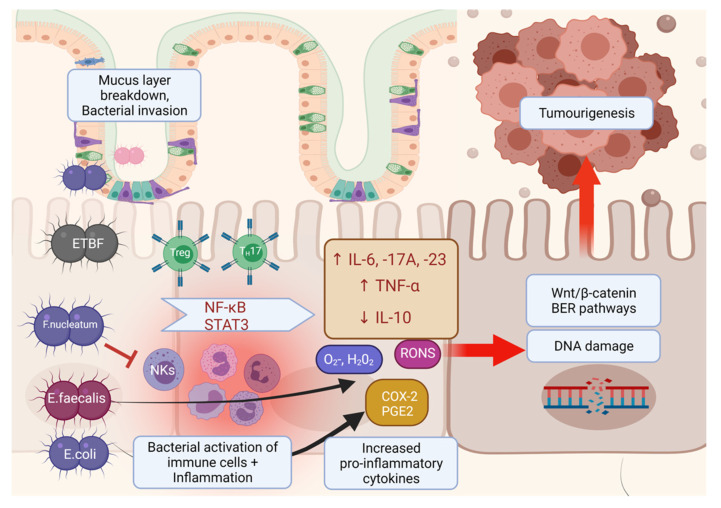
Interactions of immune response with gut microbiome facilitate CRC carcinogenesis. Dysbiosis of commensal bowel bacteria may compromise colonic epithelial barrier integrity, allowing pathogenic bacteria and their metabolites entry into the underlying stroma and promoting immune cell-mediated pro-inflammatory cytokine responses. In particular, *E.faecalis* is associated with NF-κB-mediated pro-inflammatory IL-6 and IP-10 gene expression. *F.nucleatum* is associated with IL-17A and TNF-α expression, as well as inhibiting T cell activation and natural killer cells (NKs). The presence of ETBF in the mucosa is correlated with Treg-induced Th17 production of IL-17A. Pks+-harbouring *Escherichia coli* is associated with COX-2 and PGE-2 pro-carcinoma responses. These pro-inflammatory responses can induce DNA damage and the induction of the Wnt/β-catenin and base excision repair (BER) pathways, thus increasing the risk of cell proliferation, transformation into carcinoma and subsequent metastasis.

Overall, dysbiotic bacterial degradation of the mucosal barrier, as well as its disruption of the tight junctions that lie between the lumen of the bowel and its underlying stroma, is thought to allow infiltration of bacteria and their metabolites; in turn, this process induces the recruitment of innate and adaptive immune cells [146] (Figure 2). Bacterial engagement with Toll-like receptors (TLRs) and nucleotide-binding oligomerization domain-like receptors (NLRs) on immune cells may then induce pro-inflammatory cytokines [26,147], as well as reactive oxygen and nitrogen species (RONS) [148]. This inflammation response can result in DNA damage and CIN, as well as impairment of the Wnt/β-catenin and base excision repair (BER) pathways, thus resulting in increased risk for cell proliferation, angiogenesis and metastasis [128,149].

## 7. Potential Clinical Uses of Polyp Molecular Characteristics

A greater understanding of the molecular landscapes defining premalignant colorectal lesions may reveal new opportunities to prevent CRC and alter the management of patients with a history of bowel polyps. We envisage a potential use of such molecular information to augment disease risk assessment and better optimise colonoscopy surveillance interval timing. For example, in cases where small adenomas (5–10 mm) show elevated mutational burden, such molecular data, combined with histology and clinical history, may warrant earlier colonoscopic surveillance than current guidelines suggest. Conversely, an argument could be made to extend surveillance timing in the case of a single larger polyp (e.g., 10–20 mm) that shows low mutational burden and an absence of key oncogenic drivers, such as *APC, KRAS* or *BRAF*. In a similar vein, gut microbiomes measured non-invasively from stool samples may have utility in identifying colonic polyps in patients with a history of such lesions. This would have considerable positive impacts on delivering colonoscopy services, as most screening procedures currently lead to negative findings.

How specific immune content in polyps relates to disease prognosis is still in its infancy. Even in a broader sense, immune content has yet to be utilised for cancer staging, despite its obvious importance for determining response to immunotherapies [59]. However, in CRC, use of the CD3^+^/CD8^+^ Immunoscore as a simple measure of TILs has been shown to be prognostic for disease-free survival and overall survival [60]. Immune cell content of colorectal polyps may similarly have prognostic value and alter patient surveillance management, as described above. A further idea to investigate centres on maintaining/restoring immunosurveillance capabilities through identifying new treatment targets directed at the immune infiltrate as a primary mechanism of tumour control.

## 8. Conclusions

Large-scale NGS efforts have demonstrated that subtypes of premalignant colorectal polyps contain specific genetic lesions that establish genetic instability and/or microsatellite instability. The progression of these precursor lesions to tumours occurs with the disruption of immune control and may be facilitated by interaction with gut pathogens and a state of gut microbial dysbiosis. The combination of histology, tumour mutation burden, microbiome and immune cell infiltrates will provide a clearer understanding of the molecular progression of bowel polyps to cancers, but additional research is needed to comprehensively unravel the complex intersection of molecular effectors underlying colorectal tumorigenesis. The combination of these molecular factors provides a multifaceted approach for assessing individual patient risk, with the potential to optimise colonoscopy surveillance intervals and reduce the number of negative colonoscopies conducted, without compromising patient health.

## Figures and Tables

**Table 2:** Studies examining microbiomes from human gut mucosal adenomas.

Study	Subjects	Method	Families and Genera Enriched in Adenoma	Families and Genera Depleted in Adenoma
Shen et al. [112]	4 CA, 4 HC	T-RFLP, 16S rRNA	*Dorea, Faecalibacterium*	*Bacteroides, Coprococcus*
Mira-Pascual et al. [129]	11 CA, 7 CRC, 10 HC	V1-V3 16S rRNA	*Akkermansia, Bifidobacterium, Blautia, Enterobacteriaceae, Fusobacterium*	*Faecalibacterium*
Dadkhah et al. [116]	122 CA	V1-V2 16S rRNA		*Bifidobacterium, Blautia, Faecalibacterium*
Sanarpeddy et al. [114]	33 CA, 38 HC	V1-V2 16S rRNA	*Acidovorax, Aquabacterium, Cloacibacterium, Helicobacter, Lactobacillus, Lactococcus, Pseudomonas*	*Streptococcus*
Lu et al. [130]	31 CA, 20 HC	V3-V4 16S rRNA	*Lactococcus, Pseudomonas*	*Bacillus, Enterococcus, Solibacillus*
Nugent et al. [117]	15 CA, 15 HC	MS, qPCR	*Bifidobacterium, Eubacteria*	
Mangifesta et al. [113]	12 CA	V3 16S rRNA	*Helicobacter, Lactobacillus, Klebsiella, Prevotella*	*Bacteroides, Bifidobacterium, Blautia, Escherichia-Shigella, Faecalibacterium, Romboutsia, Ruminococcaceae*
Wang et al. [115]	49 Advanced-CA, 36 HC	V4 16S rRNA	*Halomonas, Oceanospirillales, Shewanella algae*	*Bacteroides, Blautia, Coprococcus, Fusobacterium*
Kostic et al. [120]	28 CA, 27 CRC, 31 HC	qPCR	*Fusobacterium*	
McCoy et al. [119]	48 CA, 67 HC	qPCR	*Fusobacterium*	
Ito et al. [121]	343 SSA, 122 CA, 511 CRC	qPCR	*Fusobacterium nucleatum*	

CA = colorectal adenoma, CRC = colorectal carcinoma, HC = healthy control, qPCR = quantitative polymerase chain reaction, MS = mass spectrometry.
